# More Than a Text Message: Dismantling Digital Triggers to Curate Behavior Change in Patient-Centered Health Interventions

**DOI:** 10.2196/jmir.7463

**Published:** 2017-05-26

**Authors:** Frederick Muench, Amit Baumel

**Affiliations:** ^1^ Psychiatry Northwell Health Great Neck, NY United States

**Keywords:** alerts, digital triggers, text messaging, haptic triggers, reminder systems, push alerts, mHealth, mobile health, engagement, marketing, behavior change, behavioral medicine

## Abstract

Digital triggers such as text messages, emails, and push alerts are designed to focus an individual on a desired goal by prompting an internal or external reaction at the appropriate time. Triggers therefore have an essential role in engaging individuals with digital interventions delivered outside of traditional health care settings, where other events in daily lives and fluctuating motivation to engage in effortful behavior exist. There is an emerging body of literature examining the use of digital triggers for short-term action and longer-term behavior change. However, little attention has been given to understanding the components of digital triggers. Using tailoring as an overarching framework, we separated digital triggers into 5 primary components: (1) who (sender), (2) how (stimulus type, delivery medium, heterogeneity), (3) when (delivered), (4) how much (frequency, intensity), and (5) what (trigger’s target, trigger’s structure, trigger’s narrative). We highlighted key considerations when tailoring each component and the pitfalls of ignoring common mistakes, such as alert fatigue and habituation. As evidenced throughout the paper, there is a broad literature base from which to draw when tailoring triggers to curate behavior change in health interventions. More research is needed, however, to examine differences in efficacy based on component tailoring, to best use triggers to facilitate behavior change over time, and to keep individuals engaged in physical and mental health behavior change efforts. Dismantling digital triggers into their component parts and reassembling them according to the gestalt of one’s change goals is the first step in this development work.

## Introduction

Digital interventions have pervaded how we attempt to change individuals’ behavior or encourage them to engage in a health routine and have shifted the focus from patients and providers to individuals, who can now engage in self-care around the clock outside of traditional health care settings (eg, [1-3]). However, individuals’ engagement with digital interventions outside of traditional settings must compete with other events in their daily lives and fluctuating motivation to engage in effortful behavior [4]. As a result, few individuals engage proactively in mobile apps or websites across the behavior change spectrum for more than a couple of weeks without therapist contact [5].

### Digital Triggers Overview

Alerts, prompts, and cues are all stimuli designed to prompt desired actions and reactions from users. We use the term “trigger” to refer to this broad category of digital stimuli. Triggers are especially important when developing interventions aimed at engaging people in desired therapeutic activities in a nonclinical setting characterized by an abundance of competing internal and external events. Designed to prompt an internal or external reaction, these stimuli give salience to an internal or external goal by focusing an individual on the desired goal at the appropriate time. For example, placing an item near the door that we will pass on our way out or a reminder from a loved one to take an umbrella as it is about to rain is a useful environmental engineering trigger that helps us to better prepare for a desired outcome (eg, to not get wet) [6]. Digital triggers include calendars, push notifications (both standard and in-app), texts, images, haptics, and other types of digital alerts, most often delivered through a mobile phone, watch, or computer.

There is growing empirical evidence supporting the use of triggers to enhance the effectiveness of health interventions or as stand-alone health interventions [7]. Evidence suggests that digital triggers improve individuals’ engagement in interventions with a specific target, such as appointment adherence, medication adherence, homework completion, and engagement in medical and psychological treatments [8-12], especially when compared with no treatment control conditions [8]. For example, Spohr and colleagues [13] found that individuals increased their physical activity immediately after receiving a push notification compared with individuals who solely completed a mobile self-report questionnaire. Despite the potential of triggers to foster engagement, however, reviews have suggested that the evidence has been mixed due to the heterogeneity in populations, delivery types (eg, email, phone), characteristics of the target itself, and different outcome measures used [14-17].

There is a robust body of literature on the use of alerts, primarily short message service (SMS), otherwise known as text messaging, and email as an intervention in itself. Meta-analyses and reviews have highlighted the effectiveness of digital triggers as interventions for smoking cessation, alcohol use reduction, and prenatal care [8,11,18]. Interestingly, a recent meta-analysis revealed that adding other components (eg, a website) does not significantly improve pure alert-based interventions for health behaviors [8]. However, there have been mixed results regarding the efficacy of pure digital trigger-based interventions for weight loss and physical activity. Results suggest that trigger-based interventions that are designed to increase education about a topic (eg, proper prenatal care) or those that are designed to reduce impulsive responses or increase psychological well-being may be most effective because they increase goal salience in one’s natural environment at the right time [19].

### Paper Aim

Despite the promising outcomes of alert-based interventions and the prevalence of these alerts and triggers in daily life, little attention has been paid to dismantling triggers into their component parts to deepen the understanding of how they function to improve physical and mental health outcomes. While not targeting health outcomes directly, the marketing community has embraced digital triggers as core components of engagement in products and services [20]; therefore, we will draw on findings from marketing research where applicable. Our goal is to present the core components of triggers so that those developing digital health interventions can understand how to tailor those components to best engage health intervention users in the context of their environment and daily lives.

### Tailoring as the Overarching Framework

Tailoring is the method of personalizing an intervention with respect to characteristics such as content and timing to ensure an intervention’s highest level of receptivity and engagement. Most reviews and meta-analyses of text message-based interventions have indicated that tailoring interventions to the individual, particularly when also targeting a specific condition, yields larger effect sizes than not tailoring [8,11,12,19,21]. Although the health intervention itself is typically tailored according to components of behavior change theories, with readiness or importance of change being the most frequently used [5,12,22], nearly every component of a digital trigger (ie, content, frequency, interactivity) can be tailored to increase engagement.

**Figure 1:**
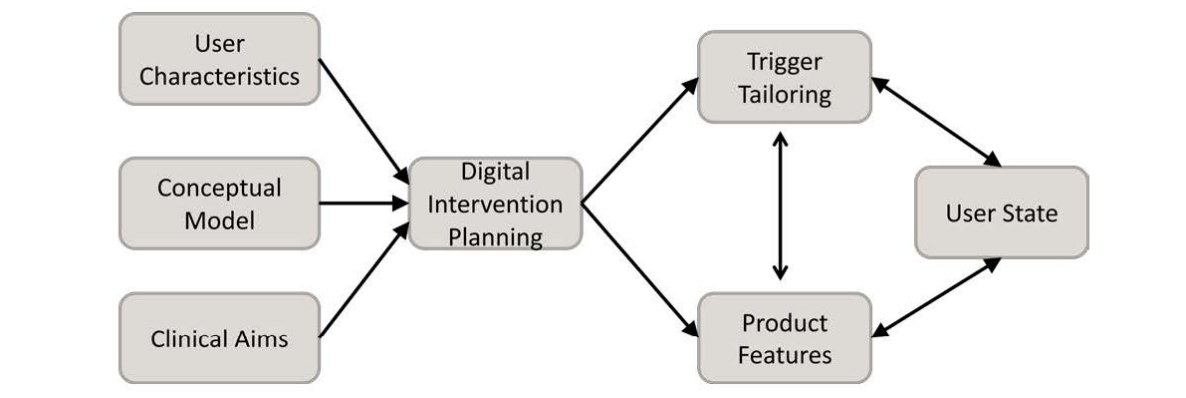
Trigger tailoring within the context of product and engagement planning and ongoing adaptation.

As mechanisms studies have revealed, tailoring can improve engagement by increasing self-referential and heuristic processing [23,24] and attention to a desired stimulus, and reducing effortful processing. Accordingly, the more that items are tailored, the more receptive an end user may become. For example, evidence from both the health and marketing literature has shown that tailoring images to end users’ demographic characteristics increases engagement [25,26] along with other strategies that match user characteristics with a persuasive strategy. Additional components, such as personalization by name, time of a trigger, end-point goal, frequency, current state, or location of receiver, can be tailored [27]. Nowhere is tailoring more evident than in the targeted advertisements on social media pages. For instance, Facebook uses 98 different data points, ranging from simple demographics (eg, gender) to life events (eg, just married) and consumer preferences (eg, preferred types of restaurants), to enable advertisers to tailor their message to specific audiences [28]. Therefore, we consider tailoring as an overall framework when developing trigger-based interventions. Below we describe each trigger component and, where possible, its relationship to other components. Figure 1 presents the concept of baseline and adaptive trigger tailoring within the context of product and engagement planning; Table 1 provides an explanatory overview of the digital trigger components described in the body of this work; and Table 2 offers a summary of our examination.

**Table 1:** Explanation of digital trigger components that can be tailored.

Component		Explanation	Examples
Who (sender)		The source of a digital trigger as it is recognized by the receiver	Automated, human, peer, sensor, organization, clinic, dog
**How**		The means by which the trigger is sent and presented	
	Stimulus type	The type of trigger presentation	Text, sound, voice, image, video, moving image, light, vibration, pressure, electrical pulse
	Delivery medium	The means by which the stimulus is delivered to the individual	Email, letter, push alert, text message, public alert system, digital banner (eg, social media)
When (just-in-time)		The best time to receive the trigger based on the recipient’s ability to pay attention and the importance of the trigger at the moment	Fixed, customized, based on user’s daily routine, context driven, data driven
How much		The frequency of triggers during the intervention phase; the context of frequency in relation to the trigger’s impact	Frequency per day, week, etc
**What**		The actual content of a trigger; should increase the recipient’s ability to *relate* to the intervention and to act in the desired way	
	Trigger’s target	The end-point goal the intervention developer is attempting to achieve in the user and the end-point goal of the user	Short-term (increase adherence, avoid forgetting), long-term (sustain engagement, avoid fatigue)
	Trigger’s structure	The arrangement of and relationships between the information parts within the medium type	Short or long message, statement, question, sound melody, vibration intensity
	Developing a story through triggers	The creation of an individual journey that relates to intervention goals within the context of the user’s life	

**Table 2:** Component summary.

Component	Highlights
Who	Messages from humans are more attended to than automated ones.
	People can attribute automated messaging to a human sender.
	The sender (or message writer) should be identified for increased credibility.
	The message source (eg, peer, loved one, clinician) should be modified based on the trigger’s message (eg, reminder, motivational note, information).
How	For stimulus type, consider the end user’s perceived burden based on the attention needed to process the information and the desired magnitude of reaction to the stimulus (eg, subtle changes in light vs electrical shock).
	Determine the delivery medium based on the strengths and weaknesses of each medium and their correspondence with the specific trigger’s context.
	Relate to the receiver’s age, communication preferences, limitations, and accessibility of the delivery method.
	Ensure trigger heterogeneity, as it will likely increase engagement.
When	Send triggers when people actually have a chance to comply with the request.
	Base triggering time on the individual’s activities and not on fixed times.
	Examine the user’s daily routine and preferences in order to send out triggers when the individual is most receptive.
	Enable users to easily customize trigger times.
How much	More is not necessarily better.
	The frequency of triggers should take into account the user’s experience of importance and readiness for change.
What	Short-term goals refer to the immediate action item embedded within the trigger; they require or prompt immediate internal or external action.
	Long-term goals refer to the sustained engagement in an intervention to guide a user toward a long-term cognitive, emotional, or behavioral shift. In this case, the user does not necessarily need to trigger an action.
	Senders typically focus on goals that they want to achieve (eg, appointment adherence), rather than what the receiver wants to achieve with a trigger (eg, feeling better after a physical therapy session). Senders are advised to be aware of this distinction as they develop triggers.
	The more relevant triggers are to the end user without manipulation, the more effective they become.
	Including links, interactivity, or human support increases the likelihood that a trigger will be attended to in the short term.
	Triggers can be seen as the adaptive control mechanism for all components of an intervention; they focus on immediate action, but also on the larger story surrounding behavior change (ie, developing a story through triggers).

## Dismantling Triggers

### Who (Sender)

There is substantial literature discussing the impact of message source credibility on increasing engagement, receptivity, and actual behavior change [29]. The source of a digital trigger can be a provider, coach, peer, friend, individual, group, blog, online publication, mobile app, sensor, or anything that can be imagined by the receiver. Subtleties embedded in the message source can alter the impact and persuasiveness of an intervention, which highlights the need to carefully consider issues of framing. For example, one study revealed that visitors to a health information webpage were more likely to take action toward change when the information was on an official webpage rather than on a blog or personal page, regardless of the content [30].

Throughout the literature, there is evidence that (1) source credibility affects recipients’ perception of delivered content [31]; (2) anonymous reviews are less credible than personally sourced reviews [32]; (3) messages from sources that are demographically closer to the receiver are more salient, and messages from people most important to the receiver are more likely to be viewed [33]; (4) messages are more persuasive if the sender is perceived as an expert before the message is sent [34,35]; and (5) digital interventions are enhanced when they are coupled with human support [36], possibly because of the combination of a human touch and the expectation of the individual’s accountability [19].

It is worth noting that the receiver’s attribution of the source might often be as powerful as the actual source of the message. Data show that people apply social rules such as politeness and reciprocity to computers [37], attribute humanistic characteristics to robots based on the latter’s responsiveness [38], and develop a therapeutic alliance with self-help programs [39]. In a study conducted in a methadone clinic, participants responded to automated text messages as if it were a social interaction; they thanked the system for sending the message, despite having been informed that the messages were automated [40]. In this case, the participants’ perception of the sender was more salient than the actual source. Perception can also be influenced by unknown source triggers where there is social reciprocity or perceived social acceptance, such as with friend request alerts or other social media notifications. Taken together, it is clear that understanding and optimizing both the user’s perception and the actual message source can have a substantial impact on trigger receptivity beyond the content. This is particularly true when the goal is to engage an end user in an actionable step that requires effort or burden.

### How

The “how” relates to the means by which the trigger is presented and sent. Here, we divide the “how” into 2 different concepts: stimulus type, which refers to how the trigger is presented, and delivery medium, which refers to how the trigger is sent.

#### Stimulus Type

The stimulus type refers to the type of trigger presentation, for example, text, voice, sound, image, moving image, light, vibration, pressure, or electrical pulse. The type of stimulus used should take the specific situation into account; there is no “one type fits all” model. For example, literature on the picture-superiority effect has shown that individuals are more likely to remember images over words [41]. Furthermore, some emerging literature in the marketing field has highlighted that images increase click rates, engagement, and sharing over text alone [42] and over other types of links [43]. The primacy of image here may be attributed to the fact that images require less effort to process, can convey more information quickly, and may be remembered for longer periods of time [41]. At the same time, images may take more time to process than simple words [44], and individual differences may mediate the relationship between the trigger type and receptivity [45].

The stimulus type affects individuals’ receptivity based on many factors that have not been sufficiently studied, yet should be taken into consideration: (1) the end user’s perceived burden based on the time and level of attention required to process the information (eg, 160 characters vs app alert icon vs long email); (2) the complexity of the interaction (eg, simple alert via vibration vs conveying a complex message); (3) the desired magnitude of reaction to the stimulus (eg, subtle changes in light vs electrical shock); and (4) individual differences in aspects such as information processing.

#### Delivery Medium

The delivery medium is the means by which the stimulus is delivered to the individual, for example, text message, phone call, push notification, or digital banner in social media apps. While emails and push alerts are most often text-based communications, these delivery methods can include any stimulus type (eg, video, images, text). Mobile phones and wearable devices have expanded the opportunity to deliver an increasing number of stimulus types beyond what was imaginable only 10 years ago. In their review of wearable solutions to improve health, Zhao and colleagues [46] highlighted that current fall-detection systems send “audible alarms, vibrations, automatic voice calls, SMS, multimedia messaging service (MMS), emails, Twitter messaging, etc,” highlighting the variability of the delivery medium. Not surprisingly, when compared with nothing, triggers delivered through almost any medium increase the individual’s engagement in the intervention, when examined independently of moderating variables that may reduce overall engagement with the intervention (eg, trigger frequency, which may lead to alert fatigue).

Compared with the scientific literature, the marketing literature unrelated to health has made considerably more effort to understand response rates and engagement based on the delivery method of the stimulus. Overall, results have suggested that text messages are more likely to be viewed than emails, are clicked significantly faster than emails and push notifications, and have higher response rates overall [47]. Several small research studies in health have found that text messages are superior to phone calls in promoting appointment attendance, and that adding other mediums to text message-based interventions does not significantly improve outcomes [8,12]. In general, compared with other mediums, text messages appear proactively on the user’s phone, are perceived as dual communication, require little programming or development experience by providers, and have the highest immediate view and response rates. At the same time, text messages require the user’s phone number, are possibly the least secure form of health information delivery, can cost the end user money, and cannot convey complex information to the user.

Email can also be triggered at specific times but carries the additional benefits of being capable of transmitting complex information, cost efficient, easily shared with multiple individuals without the need for sophisticated technology, and typically stored for years by the end user. The major downside to email is that it is often not viewed by individuals upon receipt, making it unsuitable for use with actionable tasks (eg, notification of an appointment within the hour) [48].

Push notifications triggered directly from a native app fall somewhere in between text messages and email in terms of their positives and negatives. They are cheap and easy to develop, and are part of a larger platform, which allows for a rich user experience once clicked. However, push notifications have fairly low click rates, possibly because they are not perceived as part of the human communication spectrum [49] and are embedded in nearly every app on our mobile phone, possibly causing habituation.

While the data above imply the importance of choosing the right delivery method, comparative effectiveness trials have yielded insufficient evidence as to which delivery method is best at increasing engagement in interventions. Specifically, head-to-head trials directly comparing different methods have revealed little difference in health outcomes. A systematic review of 55 studies found that response rates are generally highest for text message, followed by email and then letters [12]. However, 1 of the studies revealed that participants preferred automated voice response reminders (72%) over text message reminders (28%) [50], highlighting the heterogeneity of outcomes when it comes to delivery mediums.

In light of this heterogeneity, we recommend determining the optimal trigger medium based on the medium’s strengths and weaknesses in relation to the goals of the intervention. Should you add a digital banner or post on Instagram or Facebook, or send an email? The trigger’s delivery medium should also vary based on other factors, such as the receiver’s age (eg, email vs text message), communication preferences (eg, Snapchat vs phone call), and limitations (eg, hearing vs visual impairment); the accessibility of the delivery method (eg, mobile phone vs email); and its development and programming capacity, among others.

#### Trigger Heterogeneity

Trigger heterogeneity refers to the practice of sending triggers through different mediums, at different times and places, and from different sources. This practice is based on the multichannel and source advertising literature, which has revealed that the most effective branding and advertising comes from a range of different sources. In effect, heterogeneity in the medium (eg, text, image, video), delivery method (eg, email, phone), context (eg, home, work), time (eg, same vs varied), and source (eg, provider, friend, automated) may allow for stimulus generalization and the reduction of the habituation that results from repetition. For example, social media alerts rarely indicate the actual sender of the alert but rather that “someone” has taken an action where the receiver was salient in their mind. Sometimes this is a request for a game, while other times it is an invitation to a party. Finally, the literature on learning processes has indicated that studying information in different contexts increases information retention and recall, highlighting the opportunity to increase information processing through heterogeneity [51].

### When

The “when” refers to a certain point in time when the recipient is able to pay attention to a push notification and exert the desired amount of effort required to attain his or her goal. It derives from the concept “just-in-time,” which focuses on the individual’s receptivity within the context of ongoing tasks and daily routines [52]. People make quick decisions about how they attend to notifications as a combination of “Why am I receiving this?” and timing [53]. Although triggers can be disruptive, the recipient accepts them because they contain useful information (see “What” below). Nevertheless, adequate timing enables us to more effectively engage and support people when there is an opportunity for positive change [54,55]. Studies have repeatedly shown notifications to be effective in increasing people’s immediate engagement across a broad range of health issues such as medication uptake (eg, [56-58]), attending scheduled appointments (eg, [59,60], and brief exercise [19]; these timely reminders help people to remember to engage in beneficially perceived actions during their daily routine [61]. Most trigger timing is based on self-selected alert times and general pattern awareness (eg, medication in the morning).

People’s capacity to pay the desired amount of attention to a notification is influenced by the ongoing task they are engaged in when they receive the trigger. Mehrotra et al showed that perceived disruption increases as the complexity of an ongoing task increases [62]. In this study, mobile notifications were perceived as the most disruptive if they arrived when the user was in the middle of a task or just finishing it, and least disruptive if the user was idle or starting a new task. After work, when traveling, and at leisure were the tasks during which the users perceived notifications to be the most disruptive. However, knowing the target audience enabled the intervention developers to identify the right time windows for sending out triggers.

Similarly, MailChimp, an email marketing company that was sending billions of emails per month by 2014, analyzed the results of their time optimization system in terms of recipients’ subscription following email distributions [63]. They came to the broad conclusion that the optimal time to send emails peaked at 10:00 AM in the recipients’ own time zone between Monday and Friday (eg, work days). This finding is in line with another analysis presented by Localytics, a marketing company [64]. When categorizing the response rates for college students and bartenders, however, the optimal send time peak shifted to 1:00 PM; for neonatal nurses, who work in shifts, the results were not consistent. In effect, people tended to be more receptive to email notifications several hours after waking, but this time varied based on people’s occupation. Baumel and Schueller [65] presented similar results showing that women with perinatal mood disorders preferred to receive services late at night, when clinicians were not available, due to the fact that they were awake and had pockets of availability at that time.

These empirical findings suggest that understanding the daily routines and availability of the target audience is key to planning the trigger time, because it enables the intervention developer to make sure that the trigger is received within the right context. Research also indicates that (1) relying on reminders supports repetition, but hinders habit development; (2) evidence-based cues (eg, sending data after lunch) increase automaticity; and (3) users prefer to customize and schedule alerts to support individual aspects of their daily routine [61,66] and their relevance. For example, Muench and colleagues [67] found that individuals wanted different behavior change techniques at different moments in the change process (eg, social support messages during relapse vs cognitive reframing during craving or prelapse states).

In the future, the rise of passive data collection systems via wearable, phone-based, and external sensors will provide significantly more opportunity to deliver just-in-time adaptive contextual digital triggers to end users when they need them most. While these systems will eventually allow for greater precision in triggering users at the highest levels of receptivity based on context, timing, and data from ongoing monitoring [68,69], they require significant learning. Thus, to benefit users in the short term, intervention developers should offer end users more flexibility in personally customizing their alerts. However, customization should not sacrifice best practices of when a message might be most effective. Consequently, guiding users to apply these alerts in certain time windows based on current knowledge is recommended.

### How Much

“How much” refers to the frequency of triggers during the intervention phase. The question is whether sending out triggers more frequently increases users’ compliance with the trigger’s goal or results in trigger habituation, which eventually diminishes the reason for triggering. While “when” relates to the context of timing to maximize the trigger’s impact, “how much” relates to the impact of the trigger’s frequency on user behavior.

It is worth noting that the use of triggers in health interventions differs from the active utilization of a program. For example, there is extensive literature suggesting that, in Internet interventions, user utilization of the program results in better outcomes, implying a dose-response relationship [14,70-73]. However, this relationship can be mediated by the user’s level of motivation and persistence [74]. Triggers are different, in that sending out more triggers to the user does not equate to the user making a greater effort to engage with the intervention. Therefore, it is important for the trigger designer to determine the number and frequency of triggers that will best encourage the user’s efforts.

Determining this amount is a challenge, as data on the effect of a trigger’s frequency on health outcomes are sparse. A recent review of messaging interventions for preventive health revealed that sending more versus fewer messages resulted in better outcomes; however, this review was based on few studies, making it hard to draw conclusions about the effects of trigger intensity [11]. In a study on different interventions to increase fruit and vegetable consumption, Heimendinger and colleagues did not find any effect of tailoring from 1 mailing, but found that tailoring was effective in increasing engagement when participants received 4 mailings [75]. Another study considered variation in frequency by examining whether the frequency of text messaging feedback (1 vs 3 weekly messages) could affect smoking cessation [76]. While the timing of the first weekly text message feedback (sent to both groups) was event based—sent out to individuals when they were most receptive (ie, after sending out an assessment)—the timing of the other 2 messages, sent only to the 3-weekly messages group, did not consider users’ receptiveness; the messages were generally sent out on the 2 days following the initial message. No differences were identified between these 2 conditions. It is possible that the lack of “when” considerations for the subsequent 2 messages affected the outcomes, highlighting how the interdependency of components may affect a trigger’s effectiveness.

While research has not directly examined the habituation to triggers in patient-centered health interventions, data from related fields suggest that an uncontrolled increase in the frequency of triggers results in a decrease in the triggers’ effectiveness. Studies examining responsiveness to alerts among health providers have identified an alert fatigue: a decrease in the desired response to the alert due to its excessive appearance [77]. For example, in a literature review on physician response to drug safety alerts, van der Sijs et al found that these alerts were overridden by clinicians in 49% to 96% of cases because they receive so many irrelevant alerts [78]. When this occurs, the impact of all triggers becomes compromised. In a randomized study, Baseman et al [79] enrolled health care providers to receive public health messages and examined message content recall rates. The authors found that, for every increase of 1 local public health message per week, there was a statistically significant 41.2% decrease in the odds of the health care provider recalling the content of the study message.

Although direct empirical evidence on the impact of trigger frequency can be useful, looking more broadly into the research literature suggests that the frequency of triggers should take into account the user’s experience of importance and readiness for change. For example, people who reported that it was more important to change their drinking behavior preferred to receive more messages than those with lower importance scores [80]. Since readiness for change affects the perceived importance of the health intervention (eg, [81-84]), it results in higher adherence to treatment, engagement with treatment, and response to suggestions made by clinicians across behavioral health issues (eg, [85-87]). Accordingly, the frequency of triggers should be modified based on the perceived importance of the intervention in the user’s life along with other factors. Certainly, it is also possible to simply ask end users about their frequency preferences.

### What

#### Relating the Recipient to the Intervention

The majority of research on trigger development has focused on the actual content of the message, whether it be based on a specific theoretical underpinning (eg, gain or loss framing based on the theory of reasoned action) or content-based tailoring based on multiple factors tailored to increase engagement and outcomes, as noted above [21,88]. As noted above, content tailoring is designed to increase the recipient’s ability to *relate* to the trigger and to act in the desired way. As a result, researchers have often focused on the factors that will enable them to increase the recipient’s *relatedness* to the message [89]. For example, Kocielnik and Hsieh demonstrated that using concepts that were cognitively close to the targeted behavior (eg, for exercising: strength training, aerobics, fitness) *or* cognitively close to the message’s recipient (eg, benefits people care about or values they hold) increased recipients’ perception of the informativeness and helpfulness of the triggers, and their perception of the triggers as motivators rather than just reminders. This relatedness also resulted in higher rates of completion of the desired activity [90]. While personalization has been shown to increase engagement, it is suggested that intervention developers guide and tunnel users to adhere to best practices or avoid potentially harmful triggers created through customization. For example, in a previous message preference study, 17.1% of participants chose “aggressive messages” such as “Do you seriously think that blaming others will help you change for the better?” over a neutral-toned message [91]. Because we are at the earliest stages of trigger development, using best practices from the general health behavior change literature appears to be a useful tool in personalization and customization efforts. Unfortunately, there has been significantly less research on variations in light-, haptic-, and other nontext- or nonimage-based triggers. Therefore, it is difficult to interpret how the content and structure of a haptic- or light-based trigger may affect recipients’ engagement and goal achievement.

Despite message relevance and relatedness to the end user being identified as predictors of engagement and outcomes, many digital health interventions lean on behavior change theories (eg, social cognitive theory) as the core component of their content development. However, it is questionable whether theory-based content tailoring is more important to trigger-based interventions than increasing the recipient’s relatedness to the trigger. For example, in a recent study, we compared different types of message framing and tailoring aimed at reducing problem drinking. The results revealed little difference in drinking outcomes between theoretically distinct messaging groups (eg, gain vs loss framing vs static tailoring) [80]. Similar results were found in a recent meta-analysis, suggesting that theory may not be as important for building text message interventions as once thought [11].

In addition to the large body of literature on personalization of the trigger (eg, use of first name) and process-based tailoring (eg, motivation), we suggest 2 approaches to content framing less frequently articulated in the literature. These are the *target*, which refers to the short-term vs long-term reason or goal for which the trigger is being sent to the end user; and the *structure*, which includes the look, length, variation, or other within-trigger differentiators, and developing the behavior change narrative through a story.

#### Trigger’s Target

The target of a trigger refers to the end-point goal the intervention designer is attempting to achieve in the user and the end-point goal of the user. Senders often focus on goals that they want to achieve (eg, appointment adherence), rather than what the receiver wants to achieve as a trigger (eg, feeling better after a physical therapy session). Senders are advised to be aware of this distinction as they develop triggers. The target can be further differentiated according to long-term versus short-term goals, such as adherence to an appointment versus ongoing appointment attendance, or a 1-night drinking plan versus ongoing disease management. Understanding both short-term and long-term goals allows the message content to be tailored beyond the overall condition or goal (eg, diabetes management) and placed within a specific framework of short- versus long-term goal attainment.

##### Short-Term Goals

Short-term goals refer to the immediate action item embedded within the trigger. They are often used in marketing campaigns and appointment reminders. For example, the goal to get someone to go to a sale is often achieved by highlighting (1) that a sale exists (ie, salience), (2) that the person needs what is for sale (ie, relevance), and (3) that the sale will end soon (ie, action urgency). In the health literature, short-term goals refer to immediate actionable items, such as taking a medication or not drinking for the next several hours. Short-term triggers are designed to help the user to approach or avoid a health behavior that is actionable in the moment through cognitive reframing or behavioral plans and guidance [92]. A short-term trigger usually involves some actionable reciprocity, interaction, or potential reward that will engage the user in the moment. Including links, interactivity, or human support has been found to increase the likelihood that a trigger will be attended to in the short term [93-95].

##### Long-Term Goals

Long-term goals refer to users’ sustained engagement in an intervention to guide them toward a long-term cognitive, emotional, or behavioral shift. This is often the primary goal of chronic disease health campaigns. For example, general information on the long-term benefits of reducing alcohol intake on diabetes severity acts as a trigger to shift cognition in subtle ways. Fostering acceptance about an incurable illness is also a subtle long-term intervention target. While the intervention may result in short-term change, it does not require or prompt immediate internal or external action. Unlike with short-term targets, triggers directed toward long-term goals do not necessarily need to trigger an action on the part of the user but rather keep the end point salient over time. A recent review suggested that long-term interventions of about 6-12 months were more effective in promoting behavior change than those that were less than 6 months [11]. While little can be gleaned from these findings, they do suggest that trigger-based interventions can focus on long-term behavior change. For the most part, there are fewer examples of specific trigger types that are designed to foster long-term goal salience. While not specifically necessarily related to trigger-based interventions, an early study on virtual agents revealed that direct communication increased short-term engagement, whereas polite communication fostered long-term adherence [96,97].

##### Goal Relevance

The more that triggers are relevant to the end user without manipulation, that is, the end user agrees with the goal embedded within them, the more effective they become. Mehrotra and colleagues [62] highlighted that one of the top reasons triggers are opened is that they are relevant to the user, regardless of the senders’ goals. Indeed, although they are not mutually exclusive, receivers’ goals often have little overlap with the senders’ goals. A good example of this distinction lies in medication adherence push notifications. People are not taking medications for the sake of taking medications; they are taking medications to avoid health consequences or to feel better. Therefore, if people can avoid health consequences and feel better without medication, medication triggers become irrelevant to them. This includes the burden associated with reminding oneself to take medications, which may trigger a recurrent feeling of being ill. In light of these potential tensions, understanding the framework through which users receive the trigger and using that to create relatedness is possibly one of the most important, yet underappreciated, components of intervention development.

#### Trigger’s Structure

The structure of a trigger refers to the arrangement of and relationships between the information parts within the medium type. For example, with a text message, the structure can vary in many ways, such as long versus short messages, emoticons versus no emoticons, questions versus statements, and so on. The structure of a vibration stimulus can vary in terms of the intensity or frequency of the individual trigger. Images can vary in terms of brightness, contrast, and color. Does a sound alert employ a specific melody? Does the vibration follow a specific pattern for different message types (eg, mother vs friend) as in smartwatch alerts? These questions highlight the complex, multidimensional nature of trigger structures.

Unfortunately, little research has compared the effectiveness of different trigger structures. We recently compared users’ preferences for different short message structures, revealing that people have a range of preferences with regard to structure. For example, we found that over 90% of the sample preferred smiley emoticons, no “textese,” and proper grammar, all of which have nothing to do with the actual content of the message [91]. We also identified some moderators of structure preference. For example, compared with participants with a college degree or higher, participants with less than a college degree were more likely to prefer short messages to long messages and messages that included smiley emoticons to messages that contained no emoticons.

The structure of a trigger often varies based on system rules that are preprogrammed and learned by an individual, or based on evolutionary rules. For example, in hospital settings, triggers for specific actions often take the form of varying patterns of sounds delivered through a public alert system or vibrations in a phone or pager. The importance of alerts can also be programmed into mobile phones in a way that only the user knows the meaning of the alert. In this way, often the individual learns the trigger’s structure through slow pattern recognition. At the same time, a loud alarm alerts nearly all individuals to action without the need for overt learning and thus is often used in emergency situations. More research needs to be done to investigate how various alert structures prompt action.

#### Developing a Story Through Triggers

Triggers can be seen as actuators of an intervention or built as a component within a larger intervention framework. An actuator controls the movement or flow of system components. We think of triggers as the adaptive control mechanism for all components of an intervention that focus not only on immediate action, but also on the larger story surrounding behavior change in the context of a person’s life and changing goals. Initial baseline tailoring efforts can help build a story around salient components of an individual’s life (eg, social network), patterns of behavior (eg, usual heavy drinking times), and progress toward change in symptoms. In this way, triggers are framed to meet both long- and short-term needs and goals. A recent study on using messaging to reduce problem drinking revealed that the largest effect sizes for drinking reduction and subjective goal attainment were found among those whose messages were adapted weekly based on whether or not they met their goals for the week [80]. The adaptation of triggers in line with the users’ experiences and goals can be applied in any context to curate tailored components immediately to meet the current demands of the individual and the entire trajectory of an intervention within the context of individuals’ lives.

## Limitations

Digital triggers offer unprecedented opportunities to increase goal salience without requiring significant effort on the part of the end user. However, as noted throughout the paper, implementation efforts can backfire without understanding of the potential pitfalls of each component. Alert fatigue is a major concern among emergency and medical groups and can occur in health-based interventions as well. Subgroups of individuals in studies have reported that messages can be burdensome, too frequent, and received at inopportune times, among other complaints. Habituation or ignoring triggers is another concern that can occur with long-term trigger-based interventions. Such user responses can reduce trigger salience. In addition to these concerns over intervention effectiveness, intervention developers need to take privacy and security concerns into account. For example, text messaging is possibly the least secure method of trigger delivery, as it is stored on the phone company’s server, the messaging provider’s server, and the end user’s phone. Users should be aware of all privacy concerns prior to engaging in trigger-based adjuncts or interventions, like they would for any digital intervention.

In addition, as mentioned above in the Paper Aim paragraph, this review included papers from the marketing literature, where sometimes the goals of the intervention (eg, email subscription, special-sell) are very much different from the goals embedded within health interventions (lifetime changes needed to cope with a chronic illness). We accounted for this difference, however, by interpreting the findings from the marketing literature only within the particular context of the reviewed component (eg, when). In this way, the marketing literature could be perceived as an exploratory laboratory of specific human behaviors and reactions to triggers in the context of daily living (eg, when people are most available), which enabled us to generalize these data to the health domain.

Finally, due to the high variation in research and development methods of digital interventions that embed triggers (sometimes without clear information about component tailoring), and the broad disciplines from which data can be retrieved, we were not able to apply a systematic process to review and retrieve data (eg, systematic review). As research moves forward in this field, more evidence about the impact of trigger tailoring will emerge, and such a review would become more feasible.

## Conclusions

Fostering ongoing goal salience through adaptive tailored triggers can enhance our models of behavioral health interventions. Triggers frame the interaction with the end user as the actuator that drives the story both in the short term and over time. Understanding the nature of digital triggers and how different trigger components facilitate action is of primary importance in realizing the trigger’s potential. As evidenced throughout the paper, there is a broad literature base from which to draw when building trigger-based interventions. We recommend examining the existing literature in detail, with a special emphasis on the trigger developmental work that has primarily been done in the text messaging space [67,98]. The primary gaps in the literature appear in (1) long-term engagement appeals and (2) methods to build trigger interventions that can adapt to individuals’ current state within the larger framework of their behavior change process. Dismantling digital triggers into their component parts and reassembling them according to the gestalt of one’s change goals is the first step in this development work.

## Abbreviations

MMS: multimedia messaging service
SMS: short message service

## References

[1] Krishna S, Boren SA, Balas EA (2009). Healthcare via cell phones: a systematic review. Telemed J E Health.

[2] Norman GJ, Zabinski MF, Adams MA, Rosenberg DE, Yaroch AL, Atienza AA (2007). A review of eHealth interventions for physical activity and dietary behavior change. Am J Prev Med.

[3] Naslund JA, Marsch LA, McHugo GJ, Bartels SJ (2015). Emerging mHealth and eHealth interventions for serious mental illness: a review of the literature. J Ment Health.

[4] Baumeister RF, Vohs KD (2007). Self-regulation, ego depletion, and motivation. Social Pers Psychol Compass.

[5] Kohl LF, Crutzen R, de Vries NK (2013). Online prevention aimed at lifestyle behaviors: a systematic review of reviews. J Med Internet Res.

[6] Papies EK (2016). Health goal priming as a situated intervention tool: how to benefit from nonconscious motivational routes to health behaviour. Health Psychol Rev.

[7] Fry JP, Neff RA (2009). Periodic prompts and reminders in health promotion and health behavior interventions: systematic review. J Med Internet Res.

[8] Head KJ, Noar SM, Iannarino NT, Grant HN (2013). Efficacy of text messaging-based interventions for health promotion: a meta-analysis. Soc Sci Med.

[9] Alkhaldi G, Hamilton FL, Lau R, Webster R, Michie S, Murray E (2016). The effectiveness of prompts to promote engagement with digital interventions: a systematic review. J Med Internet Res.

[10] Guy R, Hocking J, Wand H, Stott S, Ali H, Kaldor J (2012). How effective are short message service reminders at increasing clinic attendance? A meta-analysis and systematic review. Health Serv Res.

[11] Armanasco AA, Miller YD, Fjeldsoe BS, Marshall AL (2017). Preventive health behavior change text message interventions: a meta-analysis. Am J Prev Med.

[12] De Leon E, Fuentes LW, Cohen JE (2014). Characterizing periodic messaging interventions across health behaviors and media: systematic review. J Med Internet Res.

[13] Spohr SA, Nandy R, Gandhiraj D, Vemulapalli A, Anne S, Walters ST (2015). Efficacy of SMS text message interventions for smoking cessation: a meta-analysis. J Subst Abuse Treat.

[14] Donkin L, Christensen H, Naismith SL, Neal B, Hickie IB, Glozier N (2011). A systematic review of the impact of adherence on the effectiveness of e-therapies. J Med Internet Res.

[15] Brouwer W, Kroeze W, Crutzen R, de Nooijer J, de Vries NK, Brug J, Oenema A (2011). Which intervention characteristics are related to more exposure to internet-delivered healthy lifestyle promotion interventions? A systematic review. J Med Internet Res.

[16] Christensen H, Griffiths KM, Farrer L (2009). Adherence in internet interventions for anxiety and depression. J Med Internet Res.

[17] Schubart JR, Stuckey HL, Ganeshamoorthy A, Sciamanna CN (2011). Chronic health conditions and internet behavioral interventions: a review of factors to enhance user engagement. Comput Inform Nurs.

[18] Hall AK, Cole-Lewis H, Bernhardt JM (2015). Mobile text messaging for health: a systematic review of reviews. Annu Rev Public Health.

[19] Versluis A, Verkuil B, Spinhoven P, van der Ploeg MM, Brosschot JF (2016). Changing mental health and positive psychological well-being using ecological momentary interventions: a systematic review and meta-analysis. J Med Internet Res.

[20] Rettie R, Grandcolas U, Deakins B (2005). Text message advertising: response rates and branding effects. J Targeting Meas Analysis Marketing.

[21] Webb TL, Joseph J, Yardley L, Michie S (2010). Using the internet to promote health behavior change: a systematic review and meta-analysis of the impact of theoretical basis, use of behavior change techniques, and mode of delivery on efficacy. J Med Internet Res.

[22] Lustria MLA, Noar SM, Cortese J, Van Stee SK, Glueckauf RL, Lee J (2013). A meta-analysis of web-delivered tailored health behavior change interventions. J Health Commun.

[23] Hawkins RP, Kreuter M, Resnicow K, Fishbein M, Dijkstra A (2008). Understanding tailoring in communicating about health. Health Educ Res.

[24] Chua HF, Liberzon I, Welsh RC, Strecher VJ (2009). Neural correlates of message tailoring and self-relatedness in smoking cessation programming. Biol Psychiatry.

[25] Skinner CS, Strecher VJ, Hospers H (1994). Physicians' recommendations for mammography: do tailored messages make a difference?. Am J Public Health.

[26] Kreuter MW, Sugg-Skinner C, Holt CL, Clark EM, Haire-Joshu D, Fu Q, Booker AC, Steger-May K, Bucholtz D (2005). Cultural tailoring for mammography and fruit and vegetable intake among low-income African-American women in urban public health centers. Prev Med.

[27] Galazzo L (2016). 5 push notifications strategies to increase app engagement.

[28] Dewey C (2016). 98 personal data points that Facebook uses to target ads to you.

[29] Jones L, Sinclair R, Courneya K (2003). The effects of source credibility and message framing on exercise intentions, behaviors, and attitudes: an integration of the elaboration likelihood model and prospect theory. J Appl Soc Psychol.

[30] Hu Y, Sundar SS (2010). Effects of online health sources on credibility and behavioral intentions. Commun Res.

[31] Bates BR, Romina S, Ahmed R, Hopson D (2006). The effect of source credibility on consumers' perceptions of the quality of health information on the Internet. Med Inform Internet Med.

[32] Xie H, Miao L, Kuo P, Lee B (2011). Consumers’ responses to ambivalent online hotel reviews: the role of perceived source credibility and pre-decisional disposition. Int J Hospitality Manage.

[33] Wilson EJ, Sherrell DL (1993). Source effects in communication and persuasion research: a meta-analysis of effect size. J Acad Marketing Sci.

[34] O'keefe DJ (1987). The persuasive effects of delaying identification of high‐and low‐credibility communicators: A meta‐analytic review. Commun Stud.

[35] Ward C, McGinnies E (1974). Persuasive effects of early and late mention of credible and noncredible sources. J Psychol.

[36] Schueller S, Tomasino K, Lattie E, Mohr D (2016). Human support for behavioral intervention technologies for mental health: the efficiency model.

[37] Nass C, Moon Y (2000). Machines and mindlessness: social responses to computers. J Soc Isssues.

[38] Hoffman G, Birnbaum GE, Vanunu K, Sass O, Reis HT (2014). Robot responsiveness to human disclosure affects social impression and appeal.

[39] Clarke J, Proudfoot J, Whitton A, Birch M, Boyd M, Parker G, Manicavasagar V, Hadzi-Pavlovic D, Fogarty A (2016). Therapeutic alliance with a fully automated mobile phone and web-based intervention: secondary analysis of a randomized controlled trial. JMIR Ment Health.

[40] Muench F, Adams M, McKay J, Morgenstern J, van Stolk-Cooke K (2011). Integration of text messaging for adherence to behavioral health appointments in methadone treatment.

[41] Childers TL, Houston MJ (1984). Conditions for a picture-superiority effect on consumer memory. J Consumer Res.

[42] Geerlings D (2014). Twitter engagement study: photo vs. text tweets.

[43] Redsicker P (2014). Social photos generate more engagement: new research.

[44] Snodgrass JG, McCullough B (1986). The role of visual similarity in picture categorization. J Exp Psychol Learn Mem Cogn.

[45] Fleischhauer M, Enge S, Brocke B, Ullrich J, Strobel A, Strobel A (2010). Same or different? Clarifying the relationship of need for cognition to personality and intelligence. Pers Soc Psychol Bull.

[46] Zhao F, Li M, Tsien J, Bonney W (2016). The emerging wearable solutions in mHealth. Mobile Health Technologies: Theories and Applications.

[47] Peccolo G (2015). 45 texting statistics that prove businesses need to take SMS seriously.

[48] Tolentino J (2015). SMS vs. push notification vs email: when should your app use what?.

[49] Essany M (2014). SMS marketing wallops email with 98% open rate and only 1% spam.

[50] Greaney ML, Puleo E, Sprunck-Harrild K, Bennett GG, Cunningham MA, Gillman MW, Coeling M, Emmons KM (2012). Electronic reminders for cancer prevention: factors associated with preference for automated voice reminders or text messages. Prev Med.

[51] Bouton ME, Nelson JB, Rosas JM (1999). Stimulus generalization, context change, and forgetting. Psychol Bull.

[52] Nahum-Shani I, Smith S, Tewari A, Witkiewitz K, Collins L, Spring B, Murphy S (2014). Just in time adaptive interventions (jitais): an organizing framework for ongoing health behavior support. Technical Report Number 14-126.

[53] Canel C, Rosen D, Anderson E (2000). Just-in-time is not just for manufacturing: a service perspective. Ind Manage Data Syst.

[54] Ben-Zeev D, Brenner CJ, Begale M, Duffecy J, Mohr DC, Mueser KT (2014). Feasibility, acceptability, and preliminary efficacy of a smartphone intervention for schizophrenia. Schizophr Bull.

[55] King AC, Hekler EB, Grieco LA, Winter SJ, Sheats JL, Buman MP, Banerjee B, Robinson TN, Cirimele J (2013). Harnessing different motivational frames via mobile phones to promote daily physical activity and reduce sedentary behavior in aging adults. PLoS One.

[56] Bennett JW, Glasziou PP (2003). Computerised reminders and feedback in medication management: a systematic review of randomised controlled trials. Med J Aust.

[57] Patel S, Jacobus-Kantor L, Marshall L, Ritchie C, Kaplinski M, Khurana PS, Katz RJ (2013). Mobilizing your medications: an automated medication reminder application for mobile phones and hypertension medication adherence in a high-risk urban population. J Diabetes Sci Technol.

[58] Keränen T, Liikkanen S (2013). Medication reminder service for mobile phones: an open feasibility study in patients with Parkinson's disease. Telemed J E Health.

[59] Downer SR, Meara JG, Da Costa AC, Sethuraman K (2006). SMS text messaging improves outpatient attendance. Aust Health Rev.

[60] Irigoyen MM, Findley S, Earle B, Stambaugh K, Vaughan R (2000). Impact of appointment reminders on vaccination coverage at an urban clinic. Pediatrics.

[61] Stawarz K, Cox A, Blandford A (2014). Don't forget your pill!: designing effective medication reminder apps that support users daily routines.

[62] Mehrotra A, Pejovic V, Vermeulen J, Hendley R, Musolesi M (2016). My phoneme: understanding people's receptivity to mobile notifications.

[63] Foreman J (2014). Insights from MailChimp's send time optimization system.

[64] Todd J (2016). Marketing Land.

[65] Baumel A, Schueller SM (2016). Adjusting an available online peer support platform in a program to supplement the treatment of perinatal depression and anxiety. JMIR Ment Health.

[66] Stawarz K, Cox A, Blandford A (2015). Beyond self-tracking and reminders: designing smartphone apps that support habit formation.

[67] Muench F, Weiss RA, Kuerbis A, Morgenstern J (2013). Developing a theory driven text messaging intervention for addiction care with user driven content. Psychol Addict Behav.

[68] Murray T, Hekler E, Spruijt-Metz D, Rivera D, Raij A (2016). Formalization of computational human behavior models for contextual persuasive technology.

[69] Nahum-Shani I, Hekler EB, Spruijt-Metz D (2015). Building health behavior models to guide the development of just-in-time adaptive interventions: A pragmatic framework. Health Psychol.

[70] Couper MP, Alexander GL, Zhang N, Little RJ, Maddy N, Nowak MA, McClure JB, Calvi JJ, Rolnick SJ, Stopponi MA, Cole JC (2010). Engagement and retention: measuring breadth and depth of participant use of an online intervention. J Med Internet Res.

[71] Funk KL, Stevens VJ, Appel LJ, Bauck A, Brantley PJ, Champagne CM, Coughlin J, Dalcin AT, Harvey-Berino J, Hollis JF, Jerome GJ, Kennedy BM, Lien LF, Myers VH, Samuel-Hodge C, Svetkey LP, Vollmer WM (2010). Associations of internet website use with weight change in a long-term weight loss maintenance program. J Med Internet Res.

[72] Strecher VJ, McClure J, Alexander G, Chakraborty B, Nair V, Konkel J, Greene S, Couper M, Carlier C, Wiese C, Little R, Pomerleau C, Pomerleau O (2008). The role of engagement in a tailored web-based smoking cessation program: randomized controlled trial. J Med Internet Res.

[73] Zbikowski SM, Jack LM, McClure JB, Deprey M, Javitz HS, McAfee TA, Catz SL, Richards J, Bush T, Swan GE (2011). Utilization of services in a randomized trial testing phone- and web-based interventions for smoking cessation. Nicotine Tob Res.

[74] Donkin L, Glozier N (2012). Motivators and motivations to persist with online psychological interventions: a qualitative study of treatment completers. J Med Internet Res.

[75] Heimendinger J, O'Neill C, Marcus AC, Wolfe P, Julesburg K, Morra M, Allen A, Davis S, Mowad L, Perocchia RS, Ward JD, Strecher V, Warnecke R, Nowak M, Graf I, Fairclough D, Bryant L, Lipkus I (2005). Multiple tailored messages are effective in increasing fruit and vegetable consumption among callers to the Cancer Information Service. J Health Commun.

[76] Haug S, Meyer C, Schorr G, Bauer S, John U (2009). Continuous individual support of smoking cessation using text messaging: a pilot experimental study. Nicotine Tob Res.

[77] Kesselheim AS, Cresswell K, Phansalkar S, Bates DW, Sheikh A (2011). Clinical decision support systems could be modified to reduce 'alert fatigue' while still minimizing the risk of litigation. Health Aff (Millwood).

[78] van der Sijs H, Aarts J, Vulto A, Berg M (2006). Overriding of drug safety alerts in computerized physician order entry. J Am Med Inform Assoc.

[79] Baseman JG, Revere D, Painter I, Toyoji M, Thiede H, Duchin J (2013). Public health communications and alert fatigue. BMC Health Serv Res.

[80] Muench F, van Stolk-Cooke K, Kuerbis A, Stadler G, Baumel A, Shao S, McKay JR, Morgenstern J (2017). A randomized controlled pilot trial of different mobile messaging interventions for problem drinking compared to weekly drink tracking. PLoS One.

[81] Donovan D, Rosengren D (1999). Motivation for Behavior Change and Treatment Among Substance Abusers.

[82] Laforge RG, Velicer WF, Richmond RL, Owen N (1999). Stage distributions for five health behaviors in the United States and Australia. Prev Med.

[83] Marcus BH, Rakowski W, Rossi JS (1992). Assessing motivational readiness and decision making for exercise. Health Psychol.

[84] Prochaska JO, DiClemente CC, Norcross JC (1992). In search of how people change. Applications to addictive behaviors. Am Psychol.

[85] Daley A, Duda J (2006). Self-determination, stage of readiness to change for exercise, and frequency of physical activity in young people. Eur J Sport Sci.

[86] Kerns RD, Habib S (2004). A critical review of the pain readiness to change model. J Pain.

[87] Peterson KA, Hughes M (2002). Readiness to change and clinical success in a diabetes educational program. J Am Board Fam Pract.

[88] Revere D, Dunbar PJ (2001). Review of computer-generated outpatient health behavior interventions: clinical encounters “in absentia”. J Am Med Inform Assoc.

[89] Schmid KL, Rivers SE, Latimer AE, Salovey P (2008). Targeting or tailoring? Maximizing resources to create effective health communications. Mark Health Serv.

[90] Kocielnik R, Hsieh G (2017). Send me a different message: utilizing cognitive space to create engaging message triggers.

[91] Muench F, van Stolk-Cooke K, Morgenstern J, Kuerbis AN, Markle K (2014). Understanding messaging preferences to inform development of mobile goal-directed behavioral interventions. J Med Internet Res.

[92] Kendzor DE, Shuval K, Gabriel KP, Businelle MS, Ma P, High RR, Cuate EL, Poonawalla IB, Rios DM, Demark-Wahnefried W, Swartz MD, Wetter DW (2016). Impact of a mobile phone intervention to reduce sedentary behavior in a community sample of adults: a quasi-experimental evaluation. J Med Internet Res.

[93] Wise K, Hamman B, Thorson K (2006). Moderation, response rate, and message interactivity: features of online communities and their effects on intent to participate. J Comput Mediat Commun.

[94] Sundar S, Bellur S, Oh J, Jia H, Kim HS (2016). Theoretical importance of contingency in human-computer interaction: effects of message interactivity on user engagement. Commun Res.

[95] Oh J, Sundar S, O'Brien H, Cairns P (2016). User engagement with interactive media: a communication perspective. Why Engagement Matters.

[96] Bickmore T, Schulman D, Yin L (2010). Maintaining engagement in long-term interventions with relational agents. Appl Artif Intell.

[97] Bickmore T, Mauer D, Crespo F, Brown T, Kort Y, IJsselsteijn W (2007). Persuasion, task interruption and health regimen adherence. Persuasive Technology. Lecture Notes in Computer Science.

[98] Bock BC, Rosen RK, Barnett NP, Thind H, Walaska K, Foster R, Deutsch C, Traficante R (2015). Translating behavioral interventions onto mHealth platforms: developing text message interventions for smoking and alcohol. JMIR Mhealth Uhealth.

